# Functionalized Mesoporous Thin Films for Biotechnology

**DOI:** 10.3390/mi12070740

**Published:** 2021-06-24

**Authors:** Barbara Sartori, Heinz Amenitsch, Benedetta Marmiroli

**Affiliations:** Institute of Inorganic Chemistry, Graz University of Technology, 8010 Graz, Austria; barbara.sartori@tugraz.at (B.S.); heinz.amenitsch@tugraz.at (H.A.)

**Keywords:** mesoporous thin films, silica, titania, functionalization, biotechnology, biomedicine

## Abstract

Mesoporous materials bear great potential for biotechnological applications due to their biocompatibility and versatility. Their high surface area and pore interconnection allow the immobilization of molecules and their subsequent controlled delivery. Modifications of the mesoporous material with the addition of different chemical species, make them particularly suitable for the production of bioactive coatings. Functionalized thin films of mesoporous silica and titania can be used as scaffolds with properties as diverse as promotion of cell growth, inhibition of biofilms formation, or development of sensors based on immobilized enzymes. The possibility to pattern them increase their appeal as they can be incorporated into devices and can be tailored both with respect to architecture and functionalization. In fact, selective surface manipulation is the ground for the fabrication of advanced micro devices that combine standard micro/nanofluids with functional materials. In this review, we will present the advantages of the functionalization of silica and titania mesoporous materials deposited in thin film. Different functional groups used to modify their properties will be summarized, as well as functionalization methods and some examples of applications of modified materials, thus giving an overview of the essential role of functionalization to improve the performance of such innovative materials.

## 1. Introduction

Mesoporous materials inspired by nature have been developed in the past decades, being particularly attractive because they allow for functional design and applications [1,2].

By definition, mesoporous materials present interconnected pores with a diameter in the range of 2–50 nm, which are arranged in ordered or worm-like architecture. The pores’ dimension, their arrangement, and their framework can be tuned changing the chemical composition. The whole surface can then be modified incorporating ions, functional groups or even molecules, that interact with the surface as well as with bulk material, changing its properties. In this perspective, ordered mesoporous materials play a leading role due to their versatility in terms of structural characteristics and possible applications [3]. In fact, the high surface-to-volume ratio and the interconnection of the pores, make them suitable for separation technologies, sensors, spatially defined materials for hosting cells [4], substances or reactions, or for fixation of biologically active species. More specifically, they are promising tools for biotechnology and biomedicine, in particular for in-situ treatment approaches. Recently, several studies demonstrated the multiple possibilities for biotechnological applications of mesoporous materials, thanks to their biocompatibility [5,6,7] and to their high surface area that makes them ideal vectors for drug delivery [8,9,10]. They also proved to be effective to accelerate hemostasis while preventing infections in wound sites [11].

Most of the works published so far presenting applications in biomedicine or biotechnology describe mesoporous materials in the form of nanoparticles. Nanoparticles are used because they can be injected on-site, their solubility and toxicity can be easily assessed with in vitro and in vivo tests, and they do not require to be deposited on any additional support which, in the case of biomedical applications, must be biocompatible and not lead to toxicity problems. Nevertheless, over the years both the emerging interest in the development of materials able to inhibit bacterial proliferation on surfaces such as surgical instruments, and the advantages of in-situ delivery of drugs or ions, for instance, to stimulate osteogenesis in the case of orthopaedic or dental implants, have been highlighted [12]. Mesoporous silica and titania coatings have proved to be promising appliances, since it is possible to deposit them as thin films even on irregular surfaces such as surgical screws [13]. Therefore they can be used in tissue engineering, taking advantage of their bioactivity: it was demonstrated that SiOH and TiOH groups on the surface of mesoporous silica and titania act as nucleation sites for the formation of apatite crystals when submerged in Simulated Body Fluid (SBF) [14,15], thus enhancing bone regeneration and cell growth. This opens new routes to innovative applications in regenerative medicine, allowing local administration of drugs for the repair of tissues damaged by trauma or genetic diseases, as well as for the treatment of osteoporosis [16].

For the present review, we therefore decided to focus our attention on mesoporous silica and titania thin films. In fact, among mesoporous materials, SiO_2_ is one of the most used, biocompatible substrates: its well-known and tested synthesis methods, and its predictable dissolution in a fluid with physiological compatible characteristics, [17,18,19] make it an attractive substrate in biomedicine. On the other hand, TiO_2_ shows remarkable photocatalytic activity, making it a possible choice for sterilizing coatings [13,20].

In 1992, Kresge and coworkers reported the unprecedented synthesis of well-ordered mesoporous silica materials, the widely known MCM family [21]. Shortly, their discovery was followed by the one of Antonelli and Ying from the Massachusetts Institute of Technology (MIT), who reported in 1995 the synthesis of mesoporous titanium dioxide (M-TiO_2_) [22]. Since these pioneering works, strong efforts have been put to understand the chemistry of the condensation process, as well as developing applications that could take advantage of these innovative materials. The result is a number of different synthesis protocols and a deep understanding of how these materials can be modified, to tailor them to the desired function.

Mesoporous thin films can be prepared via different approaches [23,24,25]: the most commonly used is the sol–gel method [26,27], in which the self-assembly of the organometallic species is driven by the evaporation of the solvents and consequent condensation of the surfactant, in a process known as evaporation induced self-assembly (EISA) [28]. Templating strategies allow the synthesis of hierarchically ordered materials, and give the possibility to tune the mesopores’ size and their architecture, which is a critical aspect when it comes to applications like drug delivery or surface coating. In the past few decades, several templating agents were used for the production of mesoporous material. Templating agents can be solids that act as structuring promoters for the inorganic solution and are then removed by heating, like dense or porous nanobeads [29,30,31]; alternatively, the inorganic framework condensation can be directed by the self-assembly of surfactant molecules or polymers. In this latter case, the inorganic precursor is added to a templating surfactant dissolved in a suitable solvent. Hierarchically oriented thin films are obtained from the sol–gel, which is deposited on the surface of the solid support (typically silicon or glass, but also metal alloys in case of biomedical coatings) via spray-, dip- or spin-coating. The mesostructure condenses at the evaporation of the solvents [32].

After synthesis, the film must be consolidated in order to remove the templating agent and stabilize the inorganic structure. The most commonly used consolidation process is calcination, at a temperature that typically ranges from 300 °C to 500 °C [33,34,35,36,37]. Other methods consist of the thermal treatment at low temperature followed by the extraction of the template with an adequate solvent [38,39] or via UV-O_3_ processing [40]. Furthermore, top-down techniques like UV lithography [41,42,43], Electron Beam Lithography [44] and more recently X-ray irradiation [36,45,46] allow both the consolidation and the patterning of the mesoporous structure, in order to enable the precise positioning of mesoporous areas on the substrate for the design and fabrication of novel kind of microdevices.

As an alternative approach, homogeneous layers of mesoporous nanoparticles can be prepared: once produced via EISA, the consolidated, template free nanoparticles can be diluted in a suitable solvent to obtain a colloidal suspension that is spread on solid surfaces via doctor blading [32], dip coating [47], spin coating [48], or even used to grow mesoporous thin films directly on surfaces [49]. Uniform, smooth thin films of nanoparticles can be obtained via Matrix-Assisted Pulsed Laser Evaporation (MAPLE), which is a technique derived from Pulsed Laser Deposition [50]. In this approach, a low concentration solution of polymer or organic compound (the target) dispersed in an optically absorbing solvent is frozen and subsequently irradiated with a high energy pulsed laser beam: the rapid heating causes the fast evaporation of the solvent and the resulting target ablation process induces a uniform deposition of the solute on the support surface. As most of the incident laser energy is absorbed by the solvent, the solute deposition is a soft process that occurs without photochemical or thermal decomposition of organic molecules, thus allowing the deposition of thin, uniform and smooth films of, e.g., nanoparticles loaded with sensitive molecules [51].

Mesoporous coatings of TiO_2_ and SiO_2_ have been extensively studied as supports for bone tissue regeneration [47,52,53], for controlled in situ drug release, [54,55] or for applications in the field of orthopaedics and dental implants. It was demonstrated that the surface roughness improves eucariotic cell adhesion and growth [56], while at the same time, bacterial adhesion is reduced on mesoporous surfaces [57,58], making mesoporous coatings useful tools to prevent biofilm formation. Due to their photocatalytic activity, TiO_2_ mesoporous thin films can be used as self-sterilizing material for coating medical devices [16,59] with no further modifications or molecules loading.

The anti-biofilm activity of silica is even more efficient when the mesopores are loaded with antibiotics or ions which have antibacterial properties like silver, magnesium or copper [60]. Surface functionalization can be later applied to immobilize further active molecules [61] and to change the mesoporous silica surface charge, which is related to cytotoxicity [62]. This brings forward the importance of functionalization. Functionalization is a process in which a surface is decorated by a molecule. Functionalizing the mesoporous surface, it is possible to tune its hydrophilicity/hydrophobicity [63,64], to protect the material from non-specific interactions with the surrounding environment [65,66], or to avoid binding of non-specific molecules [67], tailoring the characteristics to the foreseen applications. Additionally, the properties of bulk material can be modified [68].

Despite the great potential of these materials in nanomedicine and biotechnology, only a few studies describe the possible exploitation of functionalized mesoporous thin films. In this review, we will try to fill this gap, outlining the role of functionalization of silica and titania mesoporous thin films with small functional groups, biological molecules or ions. In particular, we will focus on applications in the field of regenerative medicine and antimicrobial coatings.

## 2. Functionalization Protocols

**Figure 1:**
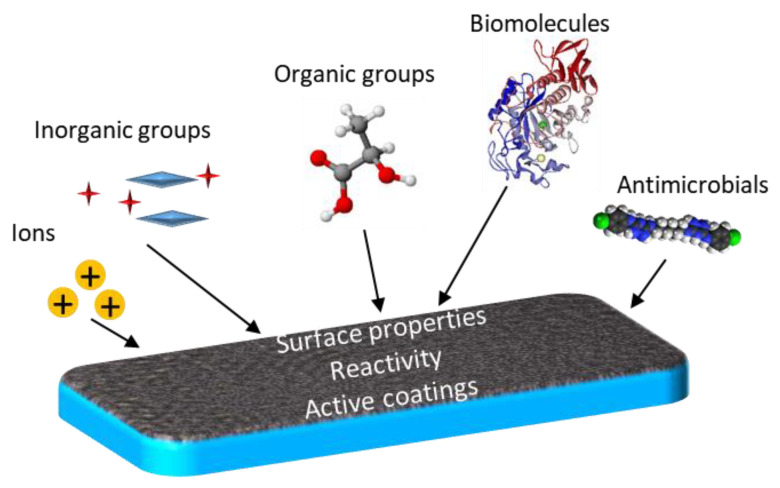
Schematic illustration of the various functionalization groups of mesoporous thin films.

Organic and inorganic groups, antimicrobial compounds [69], small biomolecules [70,71], or ions can be attached to mesoporous thin films, in order to change the material properties (Figure 1).

Functionalization can be obtained in different ways [52,65,72,73], the most common methods are co-condensation and post-grafting. In co-condensation the functionalizing group is added to the sol–gel solution and thus is distributed throughout the whole material bulk.

**Figure 2:**
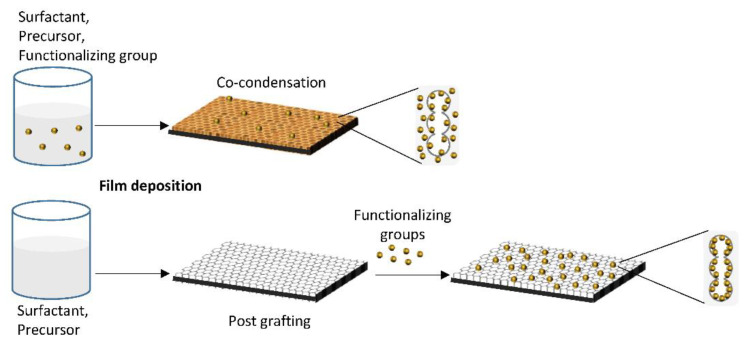
Schematic representation of mesoporous thin film functionalization. Post grafting (**bottom**) promotes the accessibility of more functional groups, with respect to co-condensation (**top**) where they are dispersed through the whole mesoporous matrix instead of the pores surface only.

In post grafting, the functionalizing groups are attached to the mesoporous surface after the film consolidation (Figure 2).

The physiochemical interaction and the interface chemistry change depending on the selected approach. The choice of the method depends on many factors which are related to each other, for example, the compatibility of the functional molecules with the mesoporous material precursor solution, or the area where the functional elements have to be present and active. Therefore the functionalization strategy should be carefully designated [74]: the simple “one pot” co-condensation is limited by the fact that the functionalizing molecule must not interfere with the self-assembly of the mesostructure, and must be resistant to the synthesis conditions. Post grafting methods allow to bypass these limitations, and to obtain a more homogeneous distribution of the functionalizing species at the mesoporous surface, as demonstrated by Calvo and coworkers. They studied the difference between post-grafting functionalization and co-condensation during silica mesoporous film synthesis, finding that in the latter case, part of the functional groups are embedded in the matrix and are not accessible for subsequent reactions: thus, the number of available functional groups on the mesoporous surface is higher for post grafting functionalized materials [75]. On the other hand, post-synthesis functionalization is limited by the dimension and the architecture of the pores, as only molecules which are small enough can diffuse into the mesoporous matrix [76].

As summarized recently by Tiemann and Weinberger [77], when different hierarchical surfaces of mesoporous materials are present, each of them can be selectively functionalized for applications in, e.g., sensing, cancer cell targeting, or drug delivery. For example, pores and surfaces can be protected with temporary ligands, or the dimension, reactivity and diffusion properties of the functionalizing agents can be used to selectively modify the material.

In the following, we will summarize some applications of functionalized silica and titania thin films, either produced via co-condensation or via post grafting. Functionalization with groups ranging from ions to biomolecules is described, aiming to emphasize the importance of surface modification of mesoporous materials for biotechnology and biomedicine.

Table 1 summarizes the main functional moieties presented in this review and their use for mesoporous silica and titania thin films.

## 3. Functional Groups

Functional groups, that involve for instance carboxy, hydroxyl, amino and thiol groups, or organometallic species, can be used to change the surface properties of mesoporous materials (Table 1). Hybrid thin films materials can be produced grafting organic moieties to silica to favour specific interactions with bioactive molecules: mercaptopropylated silanes are used to functionalize the silica surface with thiol groups, making the surface ready for further functionalization with biological molecules such as peptides by the formation of disulfide bonds [79].

Silica mesoporous nanomaterial can be functionalized with organosilanes [R-Si(OR)_3_] via post-synthetic grafting, to modify the pore’s inner surface with organic groups without affecting the mesostructure [90]. Organosilanes are used mainly to change the hydrophilic/hydrophobic character of the mesoporous surface with the addition of functional moieties such as amino- or carboxy- groups, which promote the subsequent adsorption of small molecules such as antimicrobials or enzymes, and prevent the degradation of material in physiological conditions.

In the following, we will report the properties modifications induced by functional groups, and some applications of the resulting mesoporous silica and titania thin films.

### 3.1. Change of Hydrophilicity of the Surface

A commonly used organosilane is APTES [R=(CH_2_)_3_NH_2_], which functionalizes the mesoporous surface with amino groups that make the surface more hydrophobic, and can be used to attach other organic functional groups [91].

Marmiroli and coworkers investigated the possibility to hydrate lipid membranes deposited on mesoporous silica thin films, by conveying water through the mesopores due to a change of humidity of the environment. Mesoporous films were prepared using different surfactants and consolidation treatments: thermal treatment, X-ray irradiation, and both [78].

Selected samples were functionalized by immersion in 1mM APTES solution in toluene, in a controlled atmosphere, for 12 h. The authors aimed to modify the physical properties of the surface altering the hydrophilicity of the mesoporous material and thus, tuning the water release through the mesopores which is an important aspect for lab-on-chip applications. To demonstrate the effect of functionalization on the mesostructure water uptake, the phase transition of palmitoyl-2-oleoyl-sn-glycero-3-phosphocholine (POPC) in response to hydration was evaluated. POPC was deposited on the treated mesoporous material via drop-casting, and its phase transition was evaluated with Small Angle X-ray Scattering (SAXS) changing the environmental humidity in the controlled RH ramp from 15 to 75%. For comparison, the same experiment was conducted on bare silicon wafer. The experiment results showed that lipids deposited on mesoporous material undergo hydration-driven phase transition at lower RH with respect to bare silicon, suggesting the possibility to feed water through the pores. APTES functionalized samples were less effective in conveying water to the deposited lipid, demonstrating that the functionalization with APTES reduces hydrophilicity of mesoporous materials and can be used to tailor the hydration through the pores.

Recently, the functionalization of silica mesoporous thin films to alter the wettability of the surface was obtained by vapor-phase deposition of 1H,1H,2H,2H-perfluorooctyl dimethylchlorosilane (PFODMCS) [80]. A controlled alteration of the wetting properties of mesoporous silica is useful for instance to regulate the flow of an aqueous solution in devices suitable for drug delivery. Khalil and coworkers demonstrated the possibility to tune the hydrophobicity of silica thin films produced via sol–gel EISA, changing the functionalization time. Evaluation of the surface hydrophobicity with contact angle and ellipsometry measurements, demonstrated that at a full hydrophobicity, the pores were not sterically blocked, thus they were still accessible for water loading. The access to the pores by redox molecules in acidic and alkaline conditions was also influenced by the degree of hydrophobicity of the pores, opening new sceneries for applications such as molecular separation devices and biosensors.

### 3.2. Protection against Dissolution

While titania thin films exhibit high chemical stability in biologically relevant conditions, silica thin films in aqueous environment are hydrolyzed and form silicilic acid or silicate species [19]. This progressive dissolution of the silica matrix promotes the release not only of the carried drug, but also of solubilized silica which might nucleate and accumulate randomly, posing a toxicity risk to the organism [81,92].

Functionalization with amino-groups [79,93] mercaptopropyl groups [83] or PEG [67] has also a protective effect against the dissolution of the mesoporous silica film. Fontecave and coworkers made an extensive study on the degradation of pure silica thin films in comparison with silica-zirconia, and hybrid thin films functionalized by grafting reactive alkoxysilane molecules to the mesoporous surface [81]. In an experiment performed submerging pure silica and zirconia-doped samples in PBS at biologically relevant temperatures, they demonstrated that the dissolution rate of doped silica thin film was slower than that of pure silica, and could be tuned adjusting the zirconia content. Hybrid thin films produced by co-condensation of small amounts of methylated (MTEOS) and mercaptopropyl (MPTEOS) silanes precursors showed lower dissolution rates as well, suggesting the stabilization of the Si-O bond, and the protective effect of hydrophobic functionalization groups. They also demonstrated that the concentration of amino-functionalized groups on the surface, plays a role in reducing the dissolution rate of the silica matrix.

### 3.3. Drug Loading

Surface modifications or two-steps functionalization might be necessary to adsorb enzymes or hydrophobic drugs to the mesoporous matrix.

Björk and colleagues [54] produced mesoporous silica thin films from nanoparticles via direct growth method on silicon substrates pre-treated with octa-decyltrichlorosilane [94]: this approach has the advantage that it allows to grow thin films in 3D directions [49], without the need of a flat surface to produce the thin films, which is beneficial for surgical implants, e.g., The films were functionalized with –COOH groups soaking them in a 25% water solution of carboxyethylsilanetriol di-sodium salt, in order to increase its hydrophobicity. The COOH-functionalization allowed loading the mesopores with 3,3′-dioctadecyloxacarbocyanine perchlorate (DiO), a small, yellow-fluorescent hydrophobic model drug. The amount of drug inserted and then released from the substrates was evaluated via UV-vis measurements before and after loading: the results showed that the intensity of the DiO signal at 503 nm after incubation was lowered by 15%. The stability of the immobilized drug was evaluated with the same technique on the supernatant after 24 h incubation in cell growth medium (DMEM + FCS 10%): in this case, no UV-vis signal was detected in the supernatant, confirming that the adsorbed DiO remained into the pores.

Confocal laser scanning microscopy was used to investigate the drug uptake by murine myoblast muscle cells. C2C12 cells grown on the drug-loaded mesoporous film were able to incorporate the film’s nanoparticles: once absorbed, these slowly released their drug content in the cell, as evidenced by the detectable intracellular DiO fluorescence after 4 h, and increased after 24 h of incubation.

A further experiment was performed, labelling the mesoporous nanoparticles with ATTO647N red-emitting fluorescent dye before loading them with a five-times reduced amount of DiO: this allowed to visualize clearly the fluorescence signal from both labelled silica and from the model drug DiO, thus confirming the uptake of the particles by the cells after 8 and 24 h of cells incubation.

**Figure 3:**
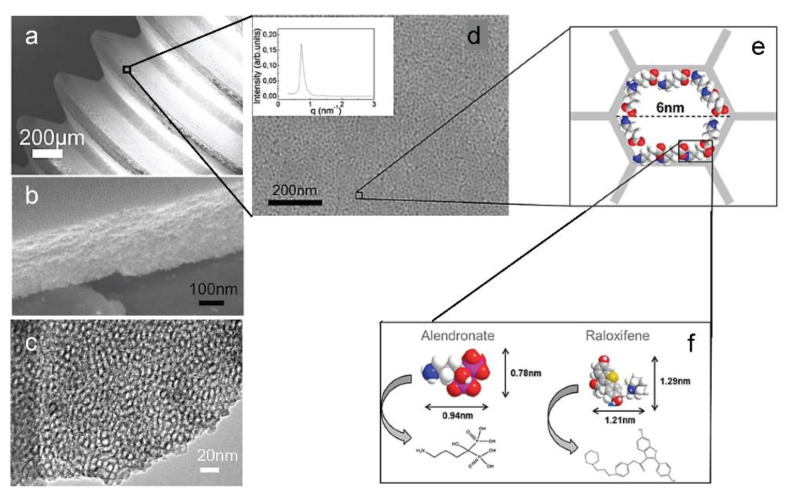
(**a**) SEM image of a screw-shaped titanium implant coated with a mesoporous TiO_2_ thin film. (**b**) Cross section of the mesoporous TiO_2_ layer. (**c**) TEM image of the TiO_2_ thin film matrix. (**d**) SEM image of mesoporous implant at a higher magnification; inset shows the synchrotron SAXS pattern for the mesoporous TiO_2_. The schematic illustration in (**e**) shows the pore diameter of 6 nm. The active substances ALN and RLX in (**f**) were absorbed into the porous structure. Adapted from N. Harmankaya et al. Acta Biomaterialia 9 (2013) 7064–7073, with permission from Elsevier.

Therapeutic molecules can be administered via mesoporous TiO_2_ thin films when a long-term, slow local drug release is desired, as in the case of bone-implant integration. EISA process allows the production of mesoporous film on non-flat surfaces, which is a great advantage for coating surgical devices. Harmankaya and colleagues performed an in-vivo study of the integration of mesoporous coated implants into the bone [82]. Osteoporosis drugs Alendronate (ALN) and Raloxifene (RLX) were immobilized in mesoporous TiO_2_ thin films prepared via EISA on custom-made titanium implant fixation screws (Figure 3).

Due to the different polarity of the two drugs, part of the implants was treated with 1% dichlorodimethylsilane (DDMS) for 30 min, in order to increase the surface hydrophobicity. The two drugs were then adsorbed to the mesopores, and the prepared implants were characterized via electron microscopy, (SEM TEM), isothermal nitrogen adsorption, diffraction (SAXS, XRD), photoelectron spectroscopy and contact angle to confirm mesoporosity, pore accessibility and hydrophobicity of the surface coating. The adsorption and release of drugs was evaluated by quartz crystal microbalance with dissipation monitoring (QCM-D) [95]. The authors observed that hydrophilic drug Alendronate loading was 30 times more effective with respect to a non-porous surface, while it was only 3 times higher for Raloxifene, which is partially explained by steric interaction of the latter drug and by the higher affinity interaction between the hydrophobic drug and the smooth hydrophobic surface. The effect of loaded and not-loaded implants on bone healing was evaluated on an animal model at 28 days from the implant, considering the mRNA expression of genetic markers for inflammatory and bone remodelling cells (TNF-α, Cathepsin K and Osteocalcin), the bio-mechanical attachment of the implant to the bone, and bone histology. Mesoporous coatings loaded with the two drugs exerted a positive effect on bone regeneration at the implant site and a stronger anchorage to the bone. These results demonstrate the capability of TiO_2_ mesoporous films as drug release tools, resulting in better implant fixation if compared to those without mesoporous coating.

## 4. Antimicrobials

Recently, the emergence of antibiotic-resistant bacterial infections raised the necessity to develop materials with amtimicrobial effects for applications in the biomedical field. In particular, biocompatible coatings with antibacterial properties are gaining more and more attention from the scientific community involved in the research and development of materials for biotechnology.

Mesoporous silica and titania thin films are promising tools as coatings to prevent bacterial colonization on surgical implants, thanks to the possibility to load the material with antibiotic drugs coupled with its low adhesive properties for biofilm growth prevention.

In a recently reported experiment, titania thin films prepared via EISA were loaded with gentamicine. The antibiofilm activity was demonstrated by evaluating the growth of *S. Aureus* on the surface of the loaded film. The results demonstrated the antibiofilm activity of the loaded material, and the prolonged release of the antibiotic through the pores.

The surface of the gentamicine-loaded mesoporous thin films was subsequently functionalized with a growth factor (human recombinant bone morphogenetic protein 2, hrBMP-2), in order to couple the antibacterial activity to improved tissue regeneration.

The preosteoblastic cell adhesion and differentiation were tested on the MC3T3-E1 osteoblast precursor cell line, showing the enhanced cell proliferation due to the presence of the growth factor attached to the mesoporous surface [84].

Functionalization with thiol groups allows for loading the mesoporous matrix with peptides or small molecules with antimicrobial activity. Recently, Izquierdo-Barba and collaborators [83] demonstrated the efficacy against bacterial colonization of mesoporous silica surfaces functionalized by co-condensation with 3-mercaptopropyl-trimethoxysilane. The film was subsequently functionalized with the small antimicrobial peptide LL-37, and with the antibacterial drug chlorexidine, and the antibacterial effect against *E. Coli* and *S. aureus* was tested, and confirmed. Functionalization did not affect the mesoporous arrangement of silica, as demonstrated by XRD measurements, thus preserving the controlled drug delivery capacity of the material. The bactericidal activity was similar after 10 months, demonstrating the stability of the mesoporous material for long-term storage. Moreover, the results showed that functionalization with the -SH group reduced the dissolution of silica in an aqueous environment, while preserving the mesostructure. In comparison with drug-loaded films, those treated with -SH showed slower drug release, demonstrating the possibility of controlling the delivery rate by functionalization.

Atefyekta and colleagues [57] synthesized mesoporous thin films of TiO_2_ with different structural characteristics, namely 4 to 7 nm pore size and film thickness from 250 to 700 nm, via EISA on titanium substrates. The deposited films were thermally treated for stabilization and template removal and selected samples were functionalized with dichlorodimethylsilane (DDMS) to increase the surface hydrophobicity and facilitate subsequent loading with low-water soluble drug daptomycin. Further film characterization showed that different pore sizes did not lead to different surface roughness, thus not affecting the anti-adhesive properties of the material. The water-accessible pore volume was evaluated via QCM-D, resulting in increased volume for higher thickness and pore size, as expected if pores are accessible in the whole film. On the other side, DDMS-treated samples showed a slightly lower pore volume, confirming the presence of the functional groups inside the pores.

The treated and non-treated samples were loaded via passive diffusion with daptomycin, vancomycin and gentamicin and used as substrates for bacterial growth, and the antimicrobial activity of the films was tested on *S. Aureus* and *P. Aeruginosa*. Both antibiotics loaded and not loaded films could reduce the bacteria adhesion, with enhanced activity in the case of hydrophobic surfaces. Antibiotic loaded material showed increased activity against bacterial film formation, as expected in presence of 7 nm mesopores. These results demonstrate the effectiveness of mesoporous titania as an antibacterial coating, which can be improved by surface functionalization.

## 5. Biofunctionalization

Enzymes can be encapsulated into mesoporous materials [96] for the production of biosensors and bioreactors, both in co-condensation processes and by adsorption to the condensed material surface. The encapsulated molecules are stable, even in environmental conditions that would normally lead to denaturation [97,98,99] such as extreme pH or temperature, and maintain their activity longer if compared to enzymes free in solution. A disadvantage is that, usually, the sol–gel condensation reaction requires the use of solvents and pH that would denature or deactivate the enzymes, so the one pot functionalization must be tailored carefully [100]. Thus, post-synthesis functionalization is preferable for these sensitive molecules.

Bellino and colleagues [86] prepared mesoporous silica thin films via EISA. The solution was deposited on glass slides via dip-coating, stabilized overnight at RT in controlled humidity and consolidated via thermal treatment. The obtained film was biofunctionalized with the enzyme α-amylase submerging the prepared substrates in enzyme solution under gentle agitation at RT. After 1 h, the excess enzyme was thoroughly rinsed with water and the activity and amount of adsorbed enzyme were evaluated. The authors demonstrated that the mesopores were accessible, that the enzyme was successfully adsorbed, and that it maintained its catalytic activity. To determine the amount of enzyme adsorbed, the pore volume was evaluated via environmental ellipsometric porosimetry (EEP) on non-treated and treated films. The latter showed a decrease in pore dimensions: according to the authors, approximately 4.3% of the available pore volume is occupied by the adsorbed enzyme, which corresponds to 1 molecule per pore. The starch-iodine colorimetric assay showed that thicker films had a significant capability to degrade starch if compared to non-functionalized silica.

Patterning and subsequent functionalization on the desired region of the mesoporous film is a challenge for the production of biosensing devices: this would allow the creation of microfluidic circuits and arrays of precise geometry, which can be selectively functionalized for the production of biosensors, as demonstrated by Doherty and colleagues [85].

Silica thin films prepared via EISA dip-coating were functionalized by submerging them in a 4.3 mM solution of APTES in toluene, and treated with 1% glutaraldehyde solution, which enabled to covalently bond the enzyme OpdA to the amino groups. The mesoporosity was confirmed with TEM. By means of an X-ray mask, selected areas of the surface were patterned with Deep X-ray Lithography to remove the functionalization, either before or after the enzyme bonding to the amino-silane. The enzyme presence after lithography was confirmed via FT-IR and its activity on the degradation of an organophosphate compound was tested. Comparison with samples without APTES functionalization demonstrated that the enzyme binding to amino-silane was much more efficient than to not-functionalized SiO_2_. A similar experiment, conducted binding a luminescent biomarker complex, confirmed the obtained results. In both cases, X-ray irradiation efficiently removed more than 80% of the bound enzyme, confirming this technique as a versatile tool for selective functionalization of mesoporous materials.

## 6. Ions

Despite the fact that silica substrates undergo biodegradation in in-vivo conditions due to the solubilization of silica matrix and the consequent formation of silicilic acid [19,101], studies have shown that they can be efficiently sterilized and still be used as bioactive coatings, having the advantage of being a source of ions necessary for cell differentiation if submerged in a solution containing inorganic ions similar to body fluid [17]. For instance, Chai and coworkers [88] used SiO_2_ mesoporous thin films as support for osteoblast-like cell growth. They studied the interaction of mesoporous silica thin films with SBF incubating spin-coated silica for different timing. They evaluated the chemical and structural characteristics of the mesoporous structure via IR spectroscopy to investigate the decay of the Si-O-Si band, X-ray diffraction, AFM to study the surface nanostructure, BET (Brunauer–Emmett–Teller) and BJT (Barrett–Joyner–Halenda) analysis to determine the surface area and the pore size respectively.

The incubation in SBF promoted the adsorption of Ca^2+^, PO_4_^3−^ and Mg^2+^ at the mesoporous surface. The positively charged ions at the surface favoured the subsequent binding of fibrinogen, a negatively charged plasma glycoprotein that facilitates cell adhesion and spreading to the surface. After an aggressive sterilization protocol which includes calcination, UV/O_3_ surface treatment, autoclave and 70% ethanol washing, the physiochemical characteristics of the SBF-treated mesoporous film were preserved, and they were successfully loaded with fibrinogen. Osteoblast-like MC3T3-E1 cells were grown on the functionalized surface for 14 days, and their differentiation degree after 10 days was evaluated testing the secreted alkaline phosphatase via ALP staining. If compared with standard tissue culture plates, the mesoporous substrate showed higher cytocompatibility and enhanced cell calcification, thus promoting the formation of bone tissue.

Several studies report the capability of mesoporous TiO_2_ to promote bone cell proliferation when functionalized with bioactive ions. Magnesium, calcium, strontium or zinc are known to promote cell differentiation of bone cell precursors and can be adsorbed on TiO_2_ mesoporous material [52,53,102,103,104]. The slow release of these ions from the mesoporous material coated on bone-implant devices such as nails or screws, for instance, promotes better bone mineralization and implant integration.

In a study to examine the implant retention and the healing effect of magnesium on bone, Galli and coworkers deposited thin films of mesoporous TiO_2_ via spin coating EISA on the surface of commercial threaded titanium implant devices [105]. After overnight stabilization at RT and subsequent thermal treatment to remove the surfactant and consolidate the mesostructure, the coated devices were immersed in an Mg^2+^ water solution for one hour. The surface of modified implants was extensively characterized with interferometry to evaluate the surface roughness, with SEM to confirm the mesoporosity and with EDX (energy-dispersive X-ray spectroscopy) to evaluate the amount of adsorbed magnesium. The in-vivo model animal study showed that mesoporous coated implants loaded with Mg^2+^ were better integrated into the healing bone, as demonstrated by the higher removal torque values observed. This suggests that magnesium-loaded mesoporous titania coatings promote bone cell adhesion [106] and better mineralization of newly formed bone at the implant site.

Akgun and collaborators demonstrated that both pure titania thin film, and functionalized Ag-TiO_2_ thin films obtained via co-condensation with AgNO_3_ exhibited bactericidal activity against *S. Epidermidis*, which was enhanced by UV irradiation [107].

The photocatalytic activity of TiO_2_ when irradiated, can be coupled with the high surface area of SiO_2_ mesoporous thin films for selective immobilization and long-term release of antimicrobial substances. Catalano and coworkers synthesized hybrid titania-silica coatings that were loaded with silver [89].

**Figure 4:**
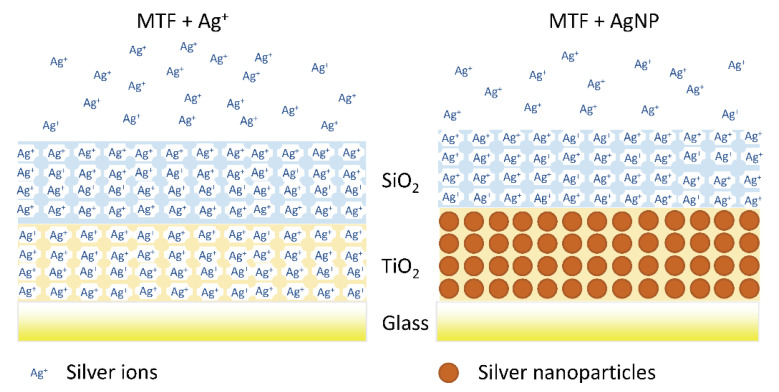
Scheme of the silver loaded silica-titania thin film (**left**) and of the film with silver nanoparticles embedded in the lower TiO_2_ layer, covered by the silica layer with Ag^+^ adsorbed (**right**). Reproduced from P.N. Catalano et al., Microporous and Mesoporous Materials 236 (2016), with permission from Elsevier.

Mesoporous thin films were produced in two steps: the first TiO_2_ layer was applied to clean microscope glass slides via dip coating. The deposition was followed by 50% RH treatment for 24 hr and thermal consolidation. Then, the second layer of SiO_2_ was deposited again via dip-coating, treated at 50% RH for 24 h and thermally consolidated. The prepared samples were submerged in 0.1 AgNO_3_ water–ethanol solutions and incubated for 1 h. The adsorbed Ag^+^ was quantified calculating the mass fraction of Ag:Ti via EDX. The authors could demonstrate the antimicrobial activity of silver nanoparticles as well as that of silver ions, thanks to the peculiar properties of both mesoporous TiO_2_ and SiO_2_. Samples immersed in AgNO_3_ were either incubated in the dark, or illuminated with a UV lamp: the UV—dependent photocatalytic activity of TiO_2_ induced in-situ photoreduction of Ag^+^ ions in the TiO_2_ layer, thus selectively confining silver nanoparticles in that mesoporous layer and leaving only silver ions in the SiO_2_ layer mesopores (Figure 4).

They demonstrated that mesoporous hybrid thin films loaded with silver ions, exhibit antimicrobial activity against *S. Aureus* and *P. Aeruginosa* comparable to that observed in the substrates loaded with Ag nanoparticles, having the advantage that the optical properties of transparent mesoporous coatings are not affected.

To circumvent the difficulties of complexing divalent ions to the mesoporous matrix, and to enhance their adsorption on the mesopores and subsequent slow release, surface-functionalized hybrid titania–silica films can be synthesized, coupling carboxylate groups via post-grafting to the surface, as demonstrated by Escobar and colleagues [87]. They produced vinyl-functionalized hybrid titania–silica-thin films and subsequently attached carboxylated groups to the surface by click-chemistry. The structure of the mesoporous thin films was characterized with SAXS, XRD and TEM which confirmed the hierarchical order and the porosity of the structure. After being submerged in an Sr^2+^ aqueous solution for 3 h, the release of ions was assessed at several time intervals, from few minutes to 7 days. Carboxylated films adsorbed double the amount of Sr^2+^ if compared to non-COOH functionalized films, and showed a more gradual Sr^2+^ release, demonstrating the effectiveness of surface functionalization for ion complexation. Both types of samples (with and without –COOH groups attached) were tested for cell viability using MC3T3-E1 pre-osteoblastic cell line from mouse calvaria: the osteoblastic proliferation was quantified, testing the activity of the enzyme alkaline phosphatase which is a well-known marker for osteoblast mineralization [108], and cells adhesion was evaluated with Confocal Laser Scanning Microscopy (CLSM) imaging. Cells grown on non-functionalized mesoporous surfaces showed better adhesion and enhanced proliferation in the first 2 days of culture, indicating the positive effect of Sr^2+^ fast release on the cell viability. Interestingly, cells cultured on -COOH functionalized surfaces showed a higher proliferation rate on the third day, and the ALP activity was significantly higher after 15 days of culture, indicating enhanced cell differentiation: in the authors’ interpretation, this is the result of the slower Sr^2+^ release in comparison with non carboxylated mesopores, which might imply a long term positive effect on bone-implant integration.

## 7. Conclusions

In this review, we summarized some applications of functionalized mesoporous silica and titania thin films for biotechnology. These innovative materials exhibit remarkable properties, such as antibacterial activities and the stimulation of cell growth, which make them particularly useful for the production of active coatings. Surface modification is the key to new functionalities, because it allows binding to the surface functional groups or molecules with potential therapeutic effects. Functionalization of mesoporous films can enable their use as nanoscaffolds for biotechnological and biomedical applications that range from bioimaging and bioanalysis to diagnosis. The large surface-to-volume ratio makes them ideal hosts for targeting molecules stored in high concentrations.

The objective of mesoporous film functionalization is to combine the pores with multiple functional groups that can be placed at desired locations on the film, and can potentially give rise to synergistic effects on host–guest interactions. Mesoporous thin films are attractive as they can be incorporated into devices and can be tailored with respect to both architecture and functionalization. The different porous systems present in hierarchically organized material can be functionalized selectively, which envisages new possibilities for the fabrication of advanced microsensors or devices that combine standard micro/nanofluids with functional materials. The endeavour here is the combination of compatible thin film preparation methods, microfabrication techniques, and functionalization protocols.

Functionalized mesoporous coatings are promising tools for those fields where long-term antibacterial activity or controlled in situ drug delivery is pursued, as well as for the development of sensors based on biologically active molecules such as enzymes.

When surface modification with biological molecules is desired, functionalization protocols might involve solvents or thermal treatment which are harmful to delicate molecules like enzymes: post-synthetic passive diffusion is the method of choice in this case, but this might not be as efficient as chemical coupling of the functional molecule to the mesoporous surface, and is limited by the pores and ligand dimension. A challenge for applications in biotechnology will be the development of functionalization methods that are efficient and at the same time would not harm the ligand. Another task would be to further develop strategies to protect the mesoporous material loaded with therapeutic molecules from degradation in biologically relevant conditions, thus avoiding the uncontrolled release of the loaded drug, or potential risks for the patient due to the matrix degradation.

The application of well-established knowledge on mesoporous thin films combined with experience in functionalization to improve the interaction with biomaterials will allow the design of new applications aimed at the clinical field. The possibility to incorporate multiple active elements in mesoporous thin films will enable tailoring the multifunctional properties to broader ranges of applications.

## Figures and Tables

**Table 1:** Summary of the functionalization groups described in the text, grouped by activity or surface modification.

Mesoporous Matrix	Primary Functionalization Group		Secondary Funct. Group	Ref	Activity
Silica	3-aminopropyl triethoxysilane (APTES)	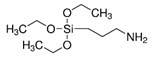		[78]	Hydrophobicity
Silica	3-aminopropyl triethoxysilane (APTES)			[79]	Hydrophobicity
Silica	1H,1H,2H,2H-perfluorooctyl dimethylchlorosilane (PFODMCS)	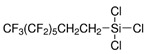		[80]	Hydrophobicity
Silica	3-mercaptopropyl triethoxysilane (MPTEOS)	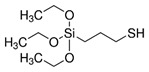		[81]	Protection Against Dissolution
Silica (hybrid film)	ZrCl_4_			[81]	Protection Against Dissolution
Silica	Triethoxymethylsilane (MTES)	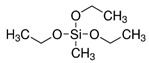		[81]	Protection Against Dissolution
Silica	3-aminopropyl triethoxysilane (APTES)			[81]	Protection Against Dissolution
Silica	PEG	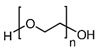		[67]	Protection Against Dissolution
Silica	Carboxyl group		3,3′-dioctadecyloxacarbocyanine perchlorate	[54]	Drug Loading
Titania	dichlorodimethylsilane (DDMS)	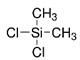	alendronate and raloxifene	[82]	Drug Loading
Titania	dichlorodimethylsilane (DDMS)		daptomycin, vancomycin, gentamicyn	[57]	Drug Loading
	3-mercaptopropyl triethoxysilane (MPTEOS)		LL-37, chlorexidine	[83]	Antimicrobials
Titania	Gentamicine		hrBMP-2	[84]	Antimicrobials
Silica	3-aminopropyltriethoxysilane (APTES)		OpdA	[85]	Bio fuctionalization
Silica	alpha-amylase			[86]	Bio functionalization
Silica-titania (hybrid)	Vinyltrimethoxysilane	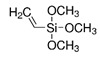	carboxyl + Sr^2+^	[87]	Ions
Silica	Ca^2+^, PO_4_^3−^, Mg^2+^ (from SBF)		fibrinogen	[88]	Ions
Titania	Mg^2+^			[52]	Ions
Titania	Ag^+^			[89]	Ions
